# Extent of Resection and Survival in IDH-Wildtype Glioblastoma: A Dual-Center Retrospective Study

**DOI:** 10.3390/medicina62020385

**Published:** 2026-02-15

**Authors:** Selami Bayram, Mustafa Serkan Alemdar, Ali Murat Tatli, Derya Kivrak Salim, Banu Ozturk, Muharrem Okan Cakir, Mustafa Ozdogan

**Affiliations:** 1 Department of Medical Oncology, Memorial Antalya Hospital, Antalya 07025, Turkey; 2 Department of Medical Oncology, Medical Park Antalya Hospital, Antalya 07160, Turkey; 3 Department of Medical Oncology, Antalya Training and Research Hospital, Antalya 07100, Turkey; 4 School of Life Science, Pharmacy and Chemistry, Kingston University, London KT1 2EE, UK

**Keywords:** Glioblastoma, gross total resection, extent of resection, IDH-wildtype, overall survival, progression-free survival, MGMT promoter methylation, Stupp protocol, treatment outcome

## Abstract

*Background and Objectives*: Glioblastoma (GBM), defined as IDH-wildtype CNS WHO grade 4, remains the most common and aggressive primary malignant brain tumor in adults. Although the extent of resection (EOR), particularly gross total resection (GTR), is considered a potentially modifiable factor, survival comparisons across surgical groups are vulnerable to selection bias and unmeasured biological confounding. We evaluated the association between GTR and survival outcomes in patients with newly diagnosed IDH-wildtype GBM in a dual-center, real-world cohort. *Materials and Methods*: We conducted a retrospective, dual-center cohort study of 100 adult patients with histopathologically confirmed GBM who underwent primary surgical resection between 2015 and 2021. GTR was defined as no measurable residual contrast-enhancing tumor on early postoperative MRI (≤72 h). All patients received adjuvant chemoradiotherapy according to the Stupp protocol. Survival was analyzed using Kaplan–Meier methods with log-rank tests and explored using univariable Cox regression analysis. Given the missing key prognostic covariates (notably MGMT promoter methylation) and the retrospective design, the analyses were reported as unadjusted and descriptive. *Results*: Of the 100 patients, 63 (63%) underwent GTR and 37 (37%) non-GTR. The GTR group had a significantly higher rate of radiologic complete response (42.9% vs. 10.8%, *p* = 0.001). However, no significant differences were observed in overall survival (OS; median 13 vs. 12 months, *p* = 0.847) or progression-free survival (PFS; 8 vs. 8 months, *p* = 0.963) between the groups in unadjusted analyses. Long-term Kaplan–Meier estimates (e.g., 5-year OS) should be interpreted cautiously due to the small number of patients at risk and potential selection and biological confounding. *Conclusions*: In this dual-center cohort, GTR was associated with improved radiologic response but not with longer OS or PFS in unadjusted analyses. These results should be considered hypothesis-generating and not interpreted as evidence against maximal safe resection. The absence of MGMT promoter methylation status, lack of volumetric EOR quantification (including non-contrast-enhancing/FLAIR disease), and lack of standardized functional outcome data substantially limited causal inference. Prospective studies integrating molecular stratification, volumetric resection metrics, and functional outcome assessments are warranted.

## 1. Introduction

Glioblastoma (GBM), defined as IDH-wildtype CNS WHO grade 4, is the most prevalent and deadliest malignant primary brain tumor in adults. Despite advances in neuro-oncology, the median overall survival seldom exceeds 15–18 months, even with optimal multimodality therapy, which typically comprises maximal safe resection followed by radiotherapy with concomitant and adjuvant temozolomide (the Stupp regimen) [1,2,3].

Among the potentially modifiable factors, the extent of resection (EOR) has consistently emerged as a key determinant of outcomes. Multiple institutional series and meta-analyses have reported longer survival with greater removal of contrast-enhancing disease while acknowledging substantial heterogeneity across cohorts and the inherent susceptibility of retrospective EOR studies to selection bias and confounding [4,5,6]. More recent studies suggest that EOR is a continuous variable and that minimizing residual contrast-enhancing volume—and, in selected cases, resection of non-contrast-enhancing (FLAIR) abnormalities—may influence outcomes within contemporary molecular frameworks [7,8,9].

The feasibility and safety of achieving GTR are strongly influenced by tumor location and proximity to the eloquent cortex, necessitating nuanced and individualized surgical planning. Sanai et al. demonstrated that survival improves once a quantitative resection threshold is surpassed, with incremental benefits at higher EOR levels [7]. Adjuncts, such as 5-aminolevulinic acid (5-ALA) fluorescence guidance and intraoperative MRI, can increase complete resection rates and/or improve short-term progression metrics by facilitating intraoperative assessment and optimization of the EOR [10,11]. Conversely, tumors contacting the subventricular zone (SVZ) are associated with poorer outcomes and may pose practical barriers to achieving GTR because of their central and infiltrative growth patterns [12]. Population-based analyses further suggest that advanced age alone should not preclude attempts at maximal safe resection when surgery can be performed safely [13]. Because “maximal safe resection” inherently reflects an onco-functional balance, the use of contemporary mapping and monitoring strategies is increasingly emphasized to expand resection while preserving neurological function, particularly in function-eloquent regions [14,15].

Against this background, we conducted a retrospective dual-center study of 100 consecutive patients with newly diagnosed GBM treated at two tertiary care institutions in Turkey, aiming to describe the association between GTR (defined on early postoperative MRI) and survival outcomes (overall and progression-free survival) in a real-world setting.

## 2. Materials and Methods

### 2.1. Study Design and Setting

We conducted a retrospective, two-center cohort study at Memorial Antalya Hospital and Antalya Education & Research Hospital (Turkey). Adult patients who underwent primary surgery for newly diagnosed glioblastoma between 2015 and 2021 were screened for inclusion.

### 2.2. Participants

The eligibility criteria included age ≥ 18 years, histopathological diagnosis consistent with glioblastoma, primary tumor surgery at a participating center, availability of an early postoperative contrast-enhanced brain MRI, and clinical follow-up at the participating center. The exclusion criteria comprised prior lower-grade glioma with malignant transformation, brain metastases/other concurrent primary brain tumors, and missing key baseline/follow-up data. In total, 121 patients were screened; 21 were excluded due to unavailable pathology records and/or missing early postoperative MRI, leaving 100 patients in the analytical cohort.

### 2.3. Pathology and Diagnostic Classification

All cases were retrospectively reclassified according to the 2021 WHO CNS classification, using the available histopathological and immunohistochemical data. Routine IDH1 R132H immunohistochemistry was performed as part of the diagnostic workup. MGMT promoter methylation was not routinely assessed in this cohort; therefore, it was not incorporated into the analyses.

### 2.4. Surgical Procedure

Patients underwent craniotomy with microsurgical tumor resection under neuronavigation, aiming for maximal safe resection. The treating neurosurgeon documented the intended extent of resection goal (GTR vs. non-GTR) based on preoperative imaging and functional considerations.

### 2.5. Imaging Protocol and Extent-of-Resection (EOR) Assessment

Early postoperative MRI was performed within 72 h after surgery and included T1-weighted post-contrast, T2, and FLAIR sequences. EOR was pragmatically operationalized as a binary variable because of the retrospective nature of the dataset and the lack of standardized volumetric segmentation across both centers. GTR was defined as no measurable residual contrast-enhancing tumor on early postoperative T1-weighted post-contrast images; non-GTR indicated visible residual contrast enhancement. Near-total (“minimal residual”) versus clearly subtotal resections could not be consistently categorized across centers using a uniform volumetric threshold, and resection of non-contrast-enhancing (FLAIR) abnormalities was not quantified. Response was assessed at the first post-CRT MRI and thereafter; the best overall response was recorded according to the RANO criteria [16]. Patients were grouped into GTR and non-GTR cohorts for analysis.

### 2.6. Adjuvant Treatment

Following surgery, patients were treated according to an intended Stupp regimen, which consists of focal radiotherapy (60 Gy in 30 fractions) with concomitant daily temozolomide (75 mg/m^2^), followed by six cycles of adjuvant temozolomide (150–200 mg/m^2^/day for five days every 28 days) [2]. Dose modifications and discontinuations adhered to institutional protocols and were recorded.

### 2.7. Data Collection and Outcomes

Demographic, clinical, radiological, and survival data were retrospectively collected from institutional electronic medical records. Clinical and MRI follow-ups were scheduled approximately every 8–12 weeks or as clinically indicated. The primary endpoint was overall survival (OS), defined as the time from the date of surgery to death from any cause or the last contact. The secondary endpoint was progression-free survival (PFS), defined as the time from surgery to radiographic progression or death, whichever occurred first. Patients without events were censored at the most recent assessment. Standardized postoperative neurological/functional outcome measures were not available in the electronic records and were therefore not analyzed; the present study focused on oncologic endpoints only.

#### 2.7.1. Statistical Analysis

Continuous variables are presented as mean (SD) or median (IQR), and categorical variables are presented as counts (percentages). Between-group comparisons were performed using the *t*-test or Mann–Whitney U test for continuous variables and χ^2^ or Fisher’s exact tests for categorical variables, as appropriate. Survival functions were estimated using Kaplan–Meier methods and compared using log-rank tests. Hazard ratios (HRs) with 95% confidence intervals were estimated using univariable Cox proportional hazards models for available covariates (age, sex, tumor side, tumor size, salvage chemotherapy, and EOR). Given the missing key prognostic variables (notably MGMT promoter methylation) and the limited capacity for stable multivariable modeling in this retrospective dataset, the analyses are reported as unadjusted and should be interpreted as descriptive rather than causal. A two-sided *p* < 0.05 was considered to be statistically significant. Analyses were performed using **IBM SPSS Statistics for Windows, Version 28.0** (IBM Corp., **Armonk, NY, USA**).

#### 2.7.2. Ethics

This study complied with the Declaration of Helsinki and was approved by the Ethics Committee of Memorial Antalya Hospital (Approval No. 311/2025; 10 March 2025). Given the retrospective design of the study, the requirement for informed consent was waived.

## 3. Results

A total of 100 patients diagnosed with glioblastoma were included in the final analysis, of whom 63 (63%) underwent gross total resection (GTR), and 37 (37%) non-GTR (residual enhancement on early postoperative MRI). The median age of the study population was 65 years (range, 28–86 years), with no statistically significant difference between the GTR and non-GTR groups (66 vs. 65 years; *p* = 0.525). The sex distribution was balanced across groups (female/male: 47/53 overall), with no significant difference between the GTR and non-GTR groups (*p* = 0.800). Tumor laterality was significantly associated with the extent of resection: right-sided tumors were more frequently resected with GTR (54% vs. 32.4%, *p* = 0.037), whereas left-sided tumors were more common in the non-GTR group (67.6%) (Table 1). This pattern may reflect functional constraints associated with dominant hemisphere or eloquent region involvement; however, detailed eloquence mapping and functional status data were not available for formal adjustment.

The median tumor size was 4 cm in both groups, ranging from 1–7.6 cm, and did not differ significantly (*p* = 0.515). Salvage chemotherapy was administered to 12% of the patients overall, without a significant difference between the groups (*p* = 0.756).

The radiological response to treatment varied significantly according to the extent of resection. Complete response (CR) was observed in 42.9% of patients in the GTR group compared with only 10.8% in the non-GTR group (*p* = 0.001). Conversely, partial response (PR) was more common in the non-GTR group (18.9% vs. 3.2%, *p* = 0.008). Notably, stable disease (SD) was only reported in the non-GTR group (21.6% vs. 0%, *p* < 0.001), whereas progressive disease (PD) occurred at similar rates in both groups (48.6% vs. 54%, *p* = 0.610). Although the overall response rate (ORR= CR + PR) was numerically higher in the GTR group (46.1% vs. 19.7%), it did not reach statistical significance (*p* = 0.107) (Table 2).

**Figure 1:**
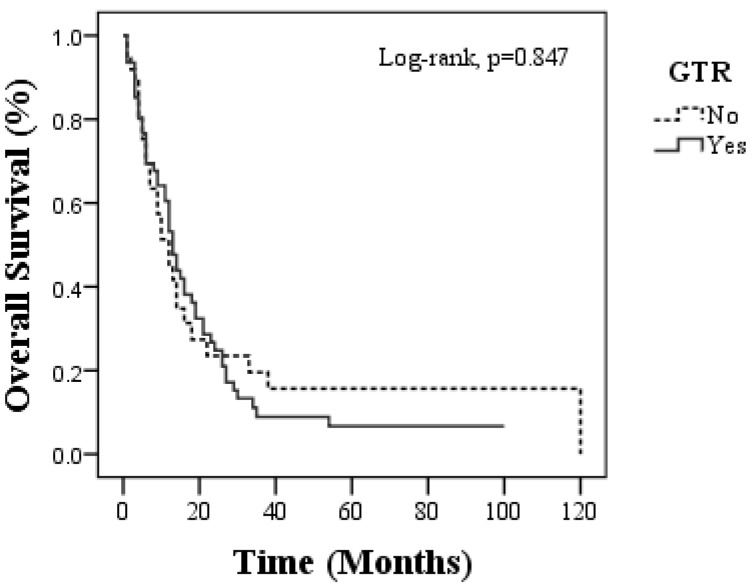
Overall survival in patients who had GTR and non-GTR.

**Figure 2:**
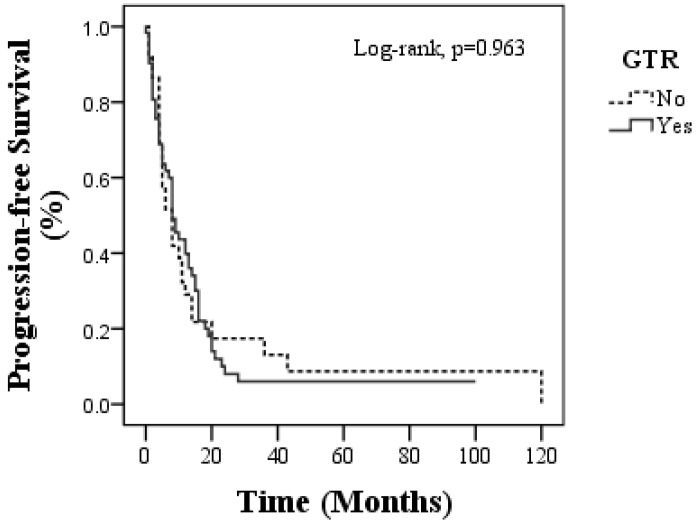
Progression-free survival in patients with GTR and non-GTR groups.

The median overall survival (OS) was 13 months (95% CI: 10.65–15.35) in the GTR group and 12 months (95% CI: 7.95–16.05) in the non-GTR group, with no statistically significant difference (*p* = 0.847). Similarly, the median progression-free survival (PFS) was 8 months in both groups (95% CI for GTR: 5.63–10.37; non-GTR: 4.81–11.19; *p* = 0.963), and the median 1-year OS rates were 53.2% for GTR and 51.3% for non-GTR, whereas the 5-year OS rates were 6.7% and 15.6%, respectively. The PFS at 1 year was higher in the GTR group (39.8% vs. 29%), but the 5-year PFS remained low across both groups (6% vs. 8.7%) (Table 2, Figure 1 and Figure 2). Long-term Kaplan–Meier estimates (e.g., 5-year OS/PFS) should be interpreted cautiously because they are influenced by small numbers at risk in the survival curve tail and censoring patterns.

In the univariate survival analysis (Table 3), none of the evaluated clinical parameters, including age, sex, tumor side, tumor size, or salvage chemotherapy, showed a statistically significant association with either OS or PFS. GTR was not significantly associated with improved OS (HR, 1.046; 95% CI, 0.655–1.672; *p* = 0.850) or PFS (HR, 1.010; 95% CI, 0.640–1.595; *p* = 0.964) (Table 4). Other variables, such as age ≥ 65 years (HR for OS: 0.840, *p* = 0.455) and tumor size > 4 cm (HR for OS: 1.180, *p* = 0.466), did not significantly affect survival outcomes.

Collectively, GTR was associated with a higher complete radiologic response rate; however, in unadjusted descriptive analyses, this did not translate into a statistically significant difference in OS or PFS in this cohort.

## 4. Discussion

In this retrospective dual-center cohort of newly diagnosed IDH-wildtype glioblastoma, GTR was associated with a higher radiologic complete response rate, whereas OS and PFS did not differ significantly between the GTR and non-GTR groups in unadjusted analyses. Because EOR is strongly linked to tumor location/eloquence and underlying tumor biology, and because key prognostic covariates were unavailable for adjustment, these findings should be interpreted as descriptive and hypothesis-generating rather than as evidence of a causal lack of benefit from maximal safe resection [2,4,5].

Several factors may account for the apparent discordance with much of the prevailing literature on this topic. IDH-wildtype glioblastoma is diffusely infiltrative, and radiologic clearance of contrast enhancement does not eliminate the microscopic disease [7,12]. More importantly, unadjusted survival comparisons are highly vulnerable to biological confounding factors. MGMT promoter methylation, a dominant prognostic and predictive biomarker in IDH-wildtype GBM treated with the Stupp regimen, was not available in our cohort [17,18]. Therefore, even a modest group imbalance in MGMT status could influence survival patterns and obscure or mimic an association between EOR and OS/PFS.

Methodological constraints related to EOR measurements are also central. We employed a pragmatic binary classification (GTR vs. non-GTR) based on early postoperative contrast-enhanced MRI, which oversimplified a continuous variable. Contemporary studies increasingly emphasize volumetric EOR thresholds and the prognostic relevance of residual contrast-enhancing volume and, in selected cases, the resection of non-contrast-enhancing (FLAIR) disease [4,6,19]. In our retrospective dataset, standardized volumetric segmentation and a consistent distinction between “minimal residual/near-total” and clearly subtotal resections were not available across both centers, which could dilute the potential dose–response relationship between EOR and survival. The higher 5-year OS rate observed in the non-GTR group should be interpreted with caution. Long-term Kaplan–Meier tail estimates are sensitive to small numbers at risk, censoring patterns, and selection biases. Accordingly, this observation should not be interpreted as evidence of the protective effect of subtotal resection but rather as a likely reflection of unmeasured biological heterogeneity and limited adjustability in this cohort.

Tumor laterality is another prognostic determinant. Right-sided tumors were more frequently resected with GTR, whereas left-sided tumors, which were often language-eloquent, underwent more non-GTR. This finding is consistent with prior reports emphasizing eloquence, rather than the hemispheric side, as the key factor limiting safe cytoreduction [14,15]. Advanced intraoperative techniques (awake mapping, navigated TMS/MEG, and tractography) can expand resection margins in eloquent regions but do not eliminate functional risks [20].

Another limitation is the absence of standardized postoperative morbidity, neurological, and functional outcome data, which prevents quantifying the clinical trade-off between cytoreduction and function. Therefore, the present study only addressed oncologic outcomes and did not allow for inferences regarding the functional consequences of pursuing GTR. The concept of “maximal safe resection” is best understood within an onco-functional balance framework, where surgical goals are individualized to preserve neurological function while maximizing cytoreduction [21,22].

When contextualized with broader evidence, most meta-analyses support the benefits of GTR. However, AbdelFatah et al. found no significant difference in OS between GTR and non-GTR (28.7 vs. 13.5 months, *p* = 0.47), underscoring the heterogeneity across cohorts [23]. Emerging strategies, such as supramaximal resection, including resection into non-contrast-enhancing FLAIR abnormalities, have demonstrated survival benefits in selected patients, especially younger individuals with good baseline performance [9,20]. Our findings are also in line with a recent systematic review and meta-analysis by Jusue-Torres et al., which specifically demonstrated improved OS and PFS associated with GTR in IDH-wildtype glioblastoma [24].

Beyond the methodology, our study provides real-world evidence from two tertiary centers in Turkey, where large-scale analyses are limited. Reporting such regional cohorts helps validate the generalizability of surgical principles across different healthcare settings. Moreover, transparently publishing negative or unexpected findings is essential to mitigate publication bias in glioblastoma research, where the benefits of GTR are often presumed to be present. Our data highlight the heterogeneity of glioblastoma biology and the importance of future prospective, molecularly stratified, multicenter studies. In addition, our cohort represents one of the largest dual-center series reported from Turkey, providing real-world evidence for a population that has been underrepresented in the literature. We believe that these data will help validate existing principles across diverse healthcare systems and contribute to novel regional insights.

### 4.1. Limitations

This study has several important limitations. First, the retrospective dual-center design and modest sample size limit generalizability and constrain inferences. Second, comprehensive molecular profiling, particularly MGMT promoter methylation, was unavailable, representing a major source of potential biological confounding in unadjusted survival comparisons. Third, survival analyses were limited to univariate models, and the results should be interpreted descriptively. Fourth, the EOR was assessed categorically without volumetric quantification and without a standardized near-total versus subtotal distinction, which may dilute the dose–response association. Fifth, standardized postoperative morbidity, neurological outcomes, and quality of life measures were not systematically captured, preventing the assessment of functional trade-offs. These limitations highlight the need for prospective, molecularly stratified studies incorporating volumetric EOR metrics and assessments of functional outcomes.

### 4.2. Clinical Implications and Future Directions

Clinically, our findings reinforce two practical implications. First, radiologic clearance of contrast-enhancing disease is an important surgical objective and is associated with an improved radiologic response in our cohort. Second, in the absence of molecular stratification and multivariate adjustment, survival patterns observed in real-world retrospective datasets should be interpreted cautiously. Our results should not be interpreted as contradicting maximal safe resection; rather, they underscore that outcomes in IDH-wildtype GBM are strongly shaped by tumor biology and selection constraints related to tumor location and functional preservation.

Future prospective studies should integrate molecular profiling, volumetric quantification of EOR (including resection of non-contrast-enhancing FLAIR regions), and systematic assessment of functional outcomes to better define the subgroups that truly benefit from aggressive resection. Moreover, our dual-center, real-world dataset from Turkey provides valuable regional evidence, and larger multicenter collaborations will be essential to validate these findings across diverse healthcare systems. This study underscores the regional novelty and broader clinical relevance of our findings.

## Figures and Tables

**Table 1:** Baseline demographic and clinical characteristics by extent of resection.

Variable		Total (n = 100)	Non-GTR (n = 37)	GTR (n = 63)	*p*
Age, years, median (range)		65 (28–86)	65 (28–75)	66 (29–86)	0.525
Gender, n(%)					0.8
	female	47 (47)	18 (48.6)	29 (46)	
	male	53 (53)	19 (51.4)	34 (54)	
Primary area, n (%)					**0.037**
	Right	46 (46)	12 (32.4)	34 (54)	
	Left	54 (54)	25 (67.6)	29 (46)	
Primary tumor size, cm, median (range)		4 (1–7.6)	4 (1–6.5)	4 (1.5–7.6)	0.515
Salvage chemotherapy, n (%)		12 (12)	5 (13.5)	7 (11.1)	0.756

**Table 2:** Radiological response and survival outcomes by extent of resection.

Variables	Non-GTR (n = 37)	GTR (n = 63)	*p*
Overall Response Rate, n (%)	11 (19.7)	29 (46.1)	0.107
Stable Disease, n (%)	8 (21.6)	0 (0)	**<0.001**
Progressive Disease, n (%)	18 (48.6)	34 (54)	0.61
Complete Response, n (%)	4 (10.8)	27 (42.9)	**0.001**
Partial Response, n (%)	7 (18.9)	2 (3.2)	**0.008**
Overall Survival, median (months)	12 (7.95–16.05)	13 (10.65–15.35)	0.847
Progression-free survival, median (months)	8 (4.81–11.19)	8 (5.63–10.37)	0.963
1-year OS, %	51.3	53.2	
5-year OS, %	15.6	6.7	
1-year PFS, %	29	39.8	
5-year PFS, %	8.7	6	

Overall Response Rate = Partial response + Complete response.

**Table 3:** Univariate analysis of factors associated with OS and PFS.

Variable	Median OS Months (95%CI)	*p*	Median PFS Months (95%CI)	*p*
**Gender**				
Male	12 (7.52–16.48)	0.982	8 (4.65–11.35)	0.674
Female	13 (10.95–15.05)		8 (4.69–11.31)	
**Age**				
<65 years	10 (4.76–15.24)	0.443	8 (4.93–11.07)	0.633
≥65 years	13 (11.08–14.93)		8 (5.59–10.41)	
**Size of tumor**				
≤4	13 (10.96–15.04)	0.454	9 (5.93–12.08)	0.555
>4	12 (7.45–16.55)		6 (3.65–8.35)	
**Salvage chemotherapy**				
No	12 (9.03–14.97)	0.193	8 (7.1–8.9)	0.864
Yes	24 (14.95–33.05)		11 (1.95–20.05)	
**Tumor side**				
Right	11 (3.9–18.11)	0.142	7 (4.01–10)	0.276
Left	14 (10.11–17.9)		10 (6.37–13.63)	
**GTR**				
No	12 (7.95–16.05)	0.847	8 (4.81–11.19)	0.963
Yes	13 (10.65–15.35)		8 (5.63–10.37)	
CI: confidence interval; OS: overall survival; PFS: progression-free survival.				

**Table 4:** Univariate Cox regression analysis of factors associated with OS and PFS.

	OS		PFS	
Variable	HR (95%CI)	*p*	HR (95%CI)	*p*
Gender (Ref: Female)	0.995 (0.637–1.555)	0.982	0.913 (0.586–1.422)	0.687
Age (Ref: <65)	0.840 (0.532–1.326)	0.455	0.901 (0.576–1.409)	0.648
Size of tumor (Ref: ≤4)	1.180 (0.756–1.843)	0.466	1.134 (0.733–1.755)	0.572
Salvage chemotherapy (Ref: no)	0.661 (0.347–1.259)	0.208	0.950 (0.512–1.761)	0.87
Tumor side (Ref: Right)	0.723 (0.462–1.130)	0.154	0.792 (0.512–1.227)	0.297
GTR (Ref: non-GTR)	1.046 (0.655–1.672)	0.85	1.010 (0.640–1.595)	0.964
CI, confidence interval; OS, overall survival; PFS, progression-free survival.				

## Data Availability

The data presented in this study are available upon request from the corresponding author. The data are not publicly available because of privacy and ethical restrictions.
